# Common Disorders and Diseases of Childhood

**Published:** 1910-09

**Authors:** 


**Common Disorders and Diseases of Childhood.** By GEORGE FREDERIC STILL, M.A., M.D. Pp. xii, 731. London: Oxford Medical Publications. 1909.

To apply the quotation from Tupper with which Dr. Still begins his book, there is much of the "clear-running wine of conviction," with but little of the "scum and lees of speculation," and the convictions expressed here are so definitely based upon facts observed as to be in their turn convincing. Dr. Still has brought to the study of pædiatrics an industry and enthusiasm which would have carried him far into the field of research even if no other qualities were added; but that which has made the explorations fruitful to the whole profession is his remarkable discernment, by which he has been enabled to reject the chaff and hold fast to the grain. This being so, it is enough to say of this book that it is a faithful expression of the author's own qualities.

It is not a systematic study of the subject, nevertheless there is not much relating to disease in children which is not alluded to in the book. In some chapters there is, perhaps, an excess of detail, for instance, in those relating to the diet of infants; but this is the inevitable outcome of Dr. Still's propensity for the registration of facts.

In many ways the book is similar to Dr. John Thomson's volume on the same subject; both are essentially clinical, in both there is a careful sifting of data, and in both the great bulk of the observations recorded have been made by the authors themselves. Some of Dr. Still's chapters are complete studies of a subject, for example, congenital syphilis; in others the sick child is described with reference to some particular symptom, as in the chapters on fever of obscure causation. These latter chapters have a peculiar freshness and charm for the jaded consumer of stereotyped text-books, and on every count the book is one to delight medical practitioners and advanced students alike. It is good to realise (in days in which depreciation of everything British is a fashionable pursuit) how much we are indebted to Great Ormond Street, to Sturges, Cheadle, Barlow, Lees, Hadden, as well as to Dr. Still and his colleagues, for our knowledge of the disorders of childhood.

The book is well bound and printed. There are a number of good pictures, and an admirable index.

